# Characteristics of Biodegradable Car Shampoos in Terms of Performance and Environmental Properties

**DOI:** 10.3390/ijms27188050

**Published:** 2026-09-10

**Authors:** Bartosz Woźniak, Marta Marzec, Mateusz Kempiński, Agata Wawrzyńczak, Izabela Nowak

**Affiliations:** 1 Department of Applied Chemistry, Faculty of Chemistry, Adam Mickiewicz University in Poznań, Uniwersytetu Poznańskiego 8, 61-614 Poznań, Poland; barwoz6@amu.edu.pl (B.W.); agata.wawrzynczak@amu.edu.pl (A.W.); 2 Department of Experimental Condensed Matter Physics, Faculty of Physics, Adam Mickiewicz University in Poznań, Uniwersytetu Poznańskiego 2, 61-614 Poznań, Poland

**Keywords:** car cosmetics, shampoos, consumer tests, surface tension, biodegradability testing, biodegradable products, chromatographic analysis, formulation stability, environmental chemistry

## Abstract

The developing chemical industry increasingly emphasizes environmentally friendly products. As consumers’ environmental awareness grows, so do their expectations of cleaning products, which must not only maintain high cleaning efficacy but also be safe for both humans and the environment. However, previous studies have not evaluated biodegradability potential alongside chemical integrity, preservation efficacy, packaging stability, interfacial performance, and consumer acceptance within a unified framework. The aim of this study was to analyze two proprietary formulations of car shampoo concentrates. Both products were assessed for basic physicochemical parameters and analyzed using chromatographic techniques, which revealed no presence of by-products or harmful substances. Subsequently, compatibility tests were conducted with PET bottles, monitoring mass loss, pH, viscosity, turbidity, and fluorescent polymer content over several months. In addition, surface tension analysis was performed using the Wilhelmy plate method, and biodegradability tests were conducted in accordance with OECD 301B in an aquatic environment, yielding 100% potential biodegradation results for both shampoos. Finally, a consumer analysis was also carried out, based on which a formulation with more favorable performance properties was selected. The integrated results provide system-level evidence that environmental performance can coexist with formulation robustness and consumer usability.

## 1. Introduction

Surfactants, particularly nonionic and amphoteric ones, reduce the surface tension of water and promote the solubilization and stabilization of active compounds in aqueous systems [1,2,3]. This function supports their widespread industrial use, including in automotive cleaning formulations [4]. Surfactants reduce surface tension by adsorbing at phase interfaces. Once the critical micelle concentration (CMC) is exceeded, micelles form and hydrophobic substances can be incorporated into their core, increasing solubilization efficiency [3,5]. At the same time, surface tension generally plateaus despite further increases in surfactant concentration [6]. Surface-tension measurements therefore provide relevant information on wetting and cleaning performance. Researchers can perform these measurements using various techniques, including the capillary, stalagmometric, hanging drop, Noüy ring, and Wilhelmy plate methods [5,7]. In car shampoos, effective surface-tension reduction improves spreading over vehicle surfaces and helps remove and solubilize hydrophobic contaminants [8].

Aqueous cleaning formulations also require adequate microbiological protection throughout manufacture, storage, and use. Contamination by bacteria, yeasts, or molds can alter product odor, color, performance, and physicochemical stability and may compromise user safety. The risk of microbiological contamination applies to many stages of a product’s life cycle: from selecting raw materials and water quality, through production and packaging, to storage and consumer use. Preservation efficacy depends on the preservative type and concentration, pH, water activity, surfactant content, and compatibility with other formulation components [9,10,11,12,13]. Challenge testing against defined microorganisms is therefore essential. PN-EN ISO 11930 provides a reference procedure and acceptance criteria for assessing a formulation’s antimicrobial protection [14].

Quality assessment of modern car shampoos must additionally confirm product composition and exclude potentially harmful contaminants or byproducts. High-performance liquid chromatography coupled with mass spectrometry (HPLC-MS) and gas chromatography coupled with mass spectrometry (GC-MS) provide complementary characterization. HPLC-MS is particularly suitable for surfactant homologs, oligomers, and isomers; for example, ethoxylated surfactants typically produce homologous series separated by 44 Da, corresponding to successive ethoxy units [15,16,17,18]. GC-MS, by contrast, supports the identification of volatile and semi-volatile constituents, including fragrance ingredients, solvents, and preservatives [19]. Together, these methods make it possible to verify whether the analyzed formulation corresponds to its intended design.

Product-packaging compatibility and environmental fate further determine product quality. Sorption, migration, permeation, packaging degradation, or formulation changes can modify product composition, stability, and safety during storage [20,21]. Similarly, biodegradability is a key parameter for assessing the safety of car shampoos in aquatic environments and for aquatic organisms. Biodegradation converts organic compounds through microbial activity into simpler products, including carbon dioxide, water, and biomass [22]. This property is especially relevant to automotive cleaning agents because product residues may enter wastewater and subsequently aquatic environments [23,24,25]. The environmental relevance is amplified by the volume of water used during vehicle washing; reported average values range from 97 L for a motorcycle and 158 L for a passenger car to 197 L for an SUV or pickup truck and 1139 L for a truck [26,27,28,29,30]. Moreover, car-wash wastewater may also contain oils, lubricants, heavy metals, and other contaminants. Surfactant biodegradability depends strongly on molecular structure, including alkyl-chain architecture and branching. It is commonly evaluated using standardized screening procedures such as OECD Guideline 301, based on endpoints including loss of dissolved organic carbon, oxygen consumption, or carbon dioxide release [31,32,33,34,35,36].

Within this context, the present study developed two concentrated car shampoo formulations (S01 and S02) and evaluated them using an integrated experimental framework comprising surface tension, HPLC-MS and GC-MS characterization, OECD 301B biodegradability, product-packaging compatibility under accelerated storage, preservative efficacy, and consumer assessment, with selected commercial shampoos used as comparators. The study therefore addresses the practical need to combine environmental performance with cleaning-related physicochemical properties, chemical quality, microbiological safety, storage stability, and user acceptance. Recent work on bio-based car shampoos has emphasized foaming and basic physicochemical/application properties [1], whereas vehicle-wash research has focused on water use, effluent composition, and treatment/reuse [26,27,28,29,30]. This leaves a formulation-level gap. To our knowledge, no study has evaluated within a single framework whether biodegradability potential can coexist with preservation efficacy, chemical integrity, product-packaging stability, interfacial performance, and consumer acceptance. The novelty lies not merely in formulating two products, but in jointly interpreting OECD 301B biodegradability, preservative efficacy, surface tension, HPLC-MS/GC-MS profiles, accelerated packaging compatibility, and consumer responses. This provides system-level evidence on environmental performance, robustness, and usability, identifying the formulation with the most balanced profile.

## 2. Results

### 2.1. Preservative Efficacy of the Shampoos

Analysis of the preservative properties of the proprietary car shampoos showed a significant reduction in microbial counts for all tested strains. For bacterial strains such as *Escherichia coli*, *Staphylococcus aureus*, and *Pseudomonas aeruginosa*, microbial counts decreased by more than 4.5 log_10_ CFU/g after 28 days of incubation. In contrast, for mold yeasts such as *Candida albicans* and *Aspergillus brasiliensis*, the reduction exceeded 3.5 log_10_ CFU/g. At the final time points, microbial counts were below the limit of detection (<10 CFU/g), and no microbial growth was observed during the study. The tests conducted indicate that the preservative system used in the proprietary car shampoos is effective against a broad spectrum of microorganisms and provides full microbiological protection, even significantly exceeding the minimum standards. The following tests show that two products with full preservative efficacy were obtained, confirmed by testing in accordance with the PN-EN ISO 11930 standard [14].

### 2.2. Biodegradability of the Developed Products

For both proprietary car shampoo formulations, the mean CO_2_ evolution reached 100% after the 28-day test period. The reported value was based on three replicate measurements. The maximum CO_2_ evolution also reached 100% for both formulations. According to the OECD 301 B criterion, a CO_2_ evolution exceeding 60% is considered evidence of ultimate biodegradation. Thus, both formulations exceeded the 60% threshold, indicating a high degree of biodegradation under OECD 301 B test conditions.

### 2.3. Surface Tension of the Car Shampoos

**Figure 1:**
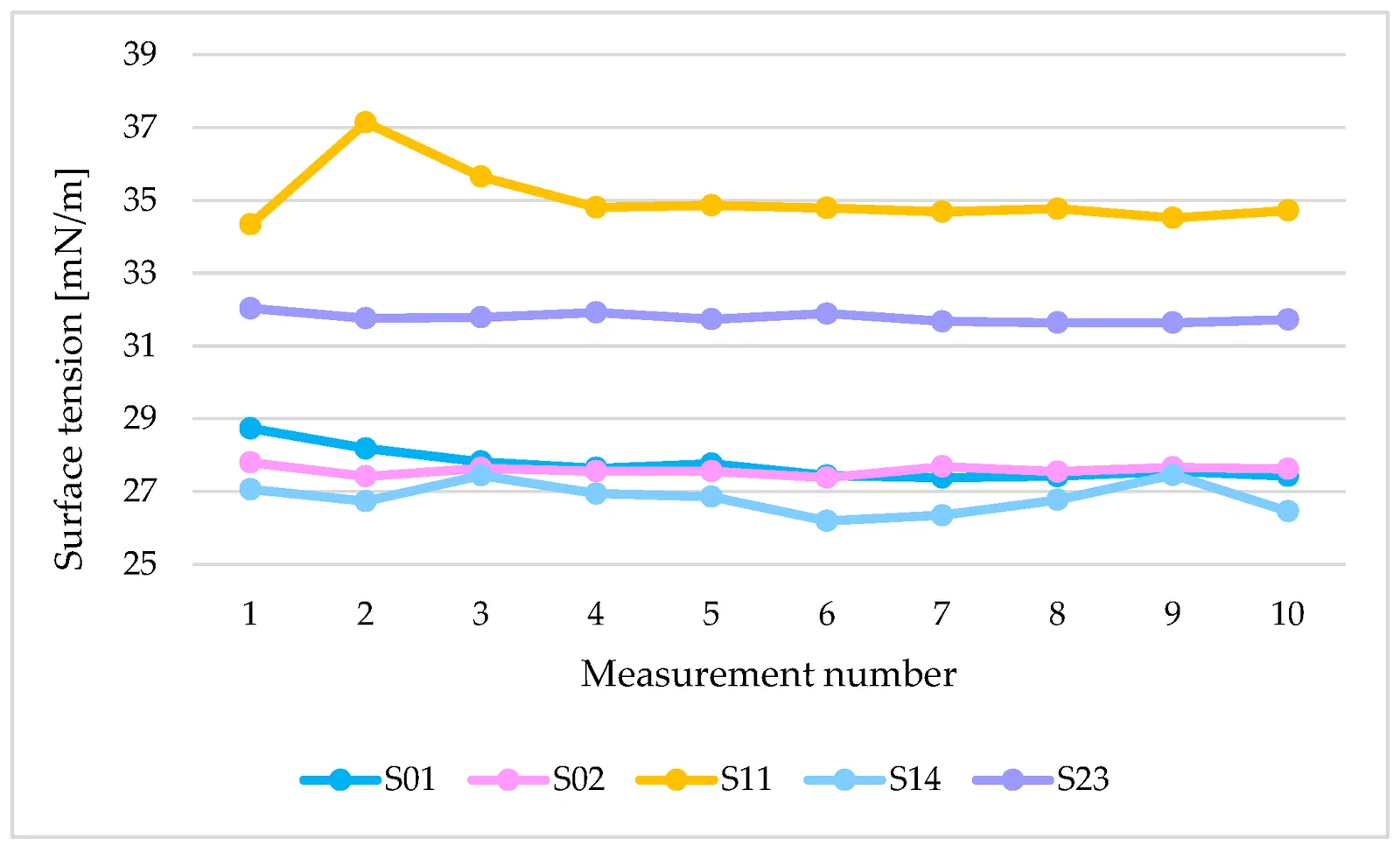
Surface tension values obtained for the tested car shampoos.

Both proprietary formulations showed significantly lower surface tension values than most other tested products at the same product concentration (5%). The results obtained for S01 and S02 were very similar. Sample S14 recorded the lowest value (26.83 ± 0.40), comparable to S01 (27.74 ± 0.41) and S02 (27.59 ± 0.12). The other shampoos tested showed higher surface tension values (31.78 ± 0.12 for S23 and 35.03 ± 0.77 for S11). The lower surface tension observed for S01 and S02 demonstrates their pronounced surface activity at the tested concentration. However, because surface tension depends not only on total surfactant concentration but also on chemical nature, surfactant interactions, and the presence of other surface-active formulation components, the measured values cannot be directly attributed to higher surfactant content. Therefore, the results should be interpreted as reflecting the overall surface activity of the formulations rather than evidence of higher surfactant concentration. All analyzed samples exhibited surface tension values lower than distilled water (Figure 1), consistent with the presence of surface-active substances in their formulations. The results show that, at the tested 5% concentration, the proprietary car shampoos effectively reduce water surface tension, with performance comparable to or better than the commercially available products tested. These findings indicate that the developed formulations exhibit pronounced surface-active properties under the investigated conditions.

### 2.4. Chromatographic Characteristics of the Tested Products

High-performance liquid chromatography coupled with mass spectrometry (HPLC-MS) was used to qualitatively screen the formulations for compounds not expected from their declared composition. In both cases, characteristic peak patterns corresponding to surfactant systems were observed, as shown in Figures S1 and S2. For shampoo S01, in ESI+ ionization mode, within the *m*/*z* range of 329–989, a regular mass increase of 44 Da was observed, corresponding to the addition of successive -[CH_2_-CH_2_-O]- units and indicating the presence of a homologous series resulting from the ethoxylation of one of the main surfactants present in both formulations. A similar effect was also observed in the ESI+ spectrum of shampoo S02, where a characteristic series of peaks differing by 44 Da in mass was noted in the *m*/*z* range of 197–945. In the spectra recorded in ESI− mode for both shampoos, the presence of regular adducts in the *m*/*z* range of 500–800 was also observed; however, their intensity was significantly lower than in the ESI+ spectra. Within the scope of the applied qualitative screening, no additional signals were observed that could not be reasonably attributed to the declared formulation ingredients or their expected homologs. The remaining signals in the MS spectra correspond to the raw materials present in the shampoo formulations and match their expected molecular weights. Because both products have very similar compositions and the HPLC-MS spectra are comparable, no additional signals were detected that could not be attributed to the formulation ingredients or their known homologs. No additional signals suggesting the formation of unexpected compounds or detectable degradation products were observed under the applied analytical conditions. We did not perform detailed analysis of signals from fragrance compositions and dyes because of their complexity and the low concentrations used in the formulations.

For gas chromatography-mass spectrometry (GC-MS) analysis, very similar results were obtained for samples dissolved in both methanol and chloroform, as shown in Figure S3. Given the similar composition of both formulations, we expected a high degree of similarity in chromatographic profiles, with any differences likely resulting from substances that distinguish the two products. In the methanol samples, an additional characteristic peak corresponding to glycerin—present in both shampoos—was observed with the expected retention time and peak shape. The GC-MS chromatograms of both shampoos show a high degree of similarity in their qualitative profiles. The dominant peaks in both chromatograms correspond to the formulation’s volatile components, mainly compounds present in the fragrance compositions. GC-MS analysis did not reveal additional volatile signals inconsistent with the expected formulation composition under the analytical conditions used. Because the fragrance composition is complex, this analysis did not aim to identify all components. The chromatographic profiles were consistent with the expected composition of both formulations. No additional chromatographic signals attributable to unexpected volatile or ionizable compounds were detected under the applied non-target screening conditions.

### 2.5. Product-Packaging Compatibility

During the compatibility analyses of proprietary automotive products and their packaging, an organoleptic evaluation of the tested products was also conducted after a 3-month testing period. Both the S01 and S02 shampoos retained their original cleansing and foaming properties after 3 months of storage under significantly elevated temperatures. The fragrance compositions also did not change their properties and remained consistent with the reference samples. The only difference noted in the tested product samples was a slight color change in both developed products, which remained acceptable in the final analysis. Because the formulations use highly biodegradable raw materials, including the coloring systems, this effect was expected. Biodegradable dyes are characterized by low stability and the potential for color changes under storage conditions (changes in pH, temperature, UV radiation). However, this effect does not pose a significant risk to the final product or the user and does not occur when the product is stored at room temperature.

#### 2.5.1. Changes in pH

**Figure 2:**
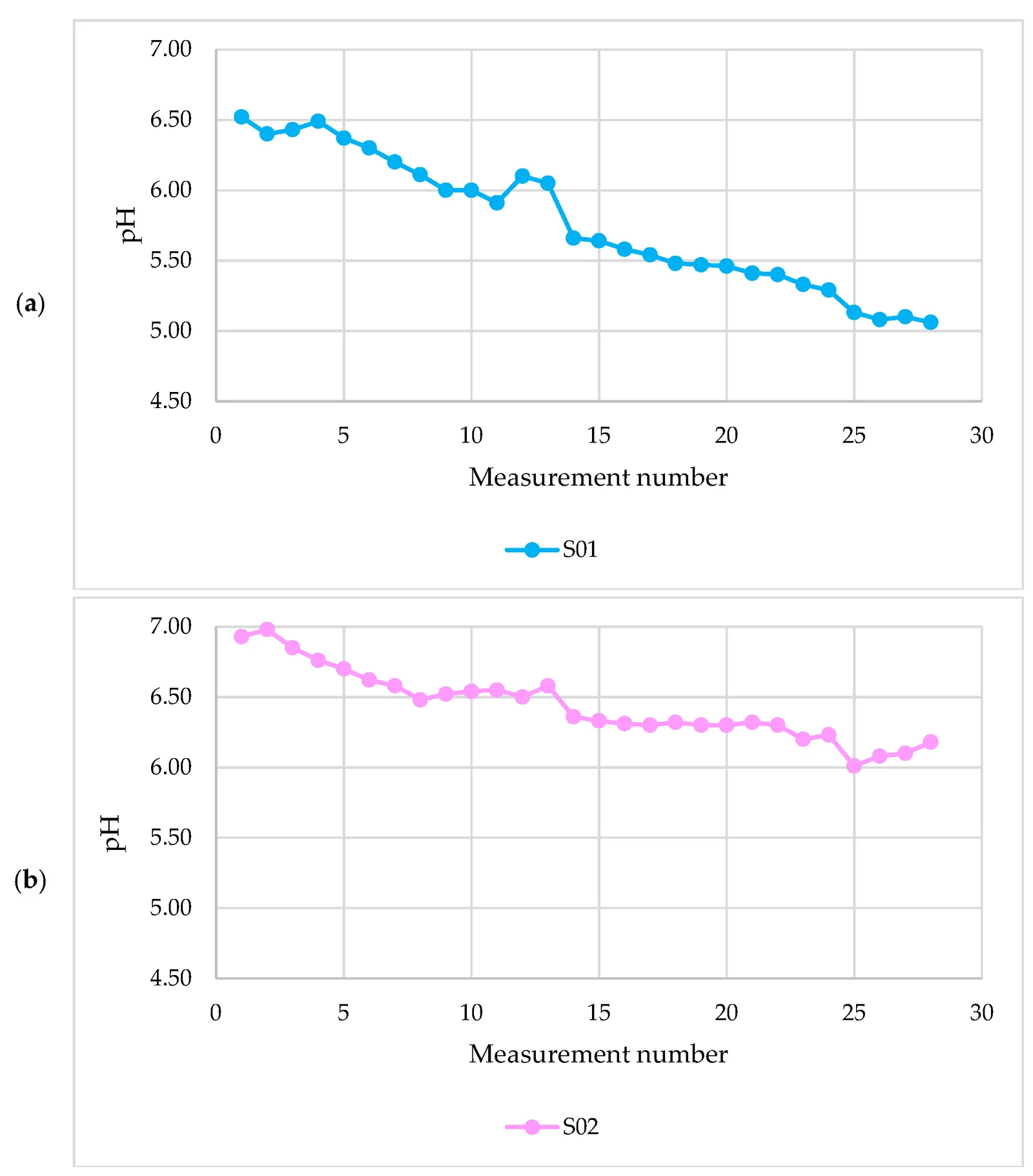
Changes in pH over time for shampoos: (**a**) S01; (**b**) S02.

Regarding pH changes, both shampoos showed a gradual decrease. For shampoo S02, the decrease was slight, and both the initial and final values remained at 6. For shampoo S01, the final value dropped below 6, reaching approximately 5.1. The final values from the last measurements were similar, suggesting these parameters would remain stable in subsequent testing periods. At the same time, for both products, the final values fell within the accepted measurement error (±1.5). Given the products’ readily biodegradable formula, a slight decrease in pH was expected. The observed differences in pH values, shown in Figure 2, are not alarming and fall within the range typical for automotive cosmetics.

#### 2.5.2. Changes in Viscosity

**Figure 3:**
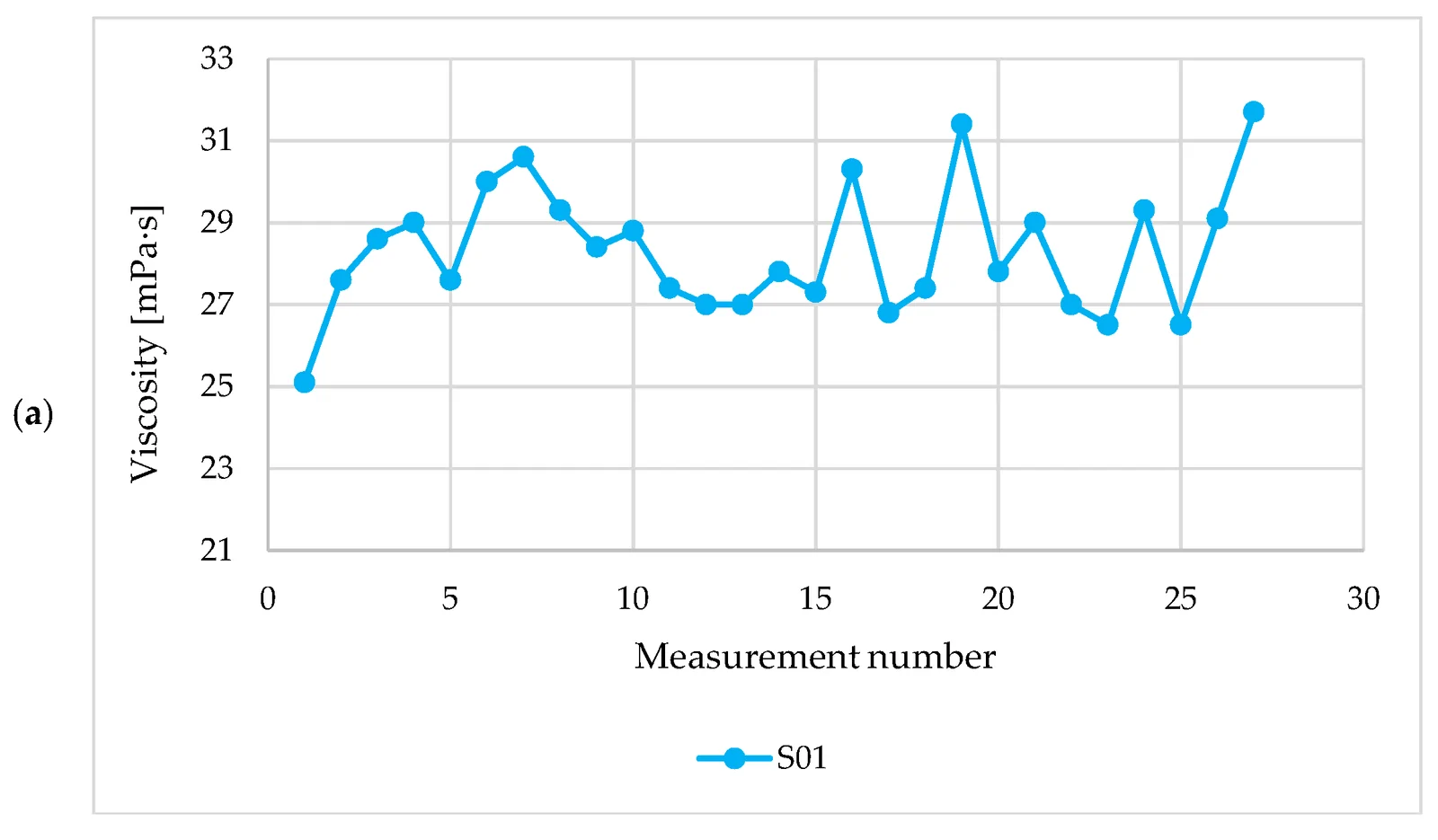

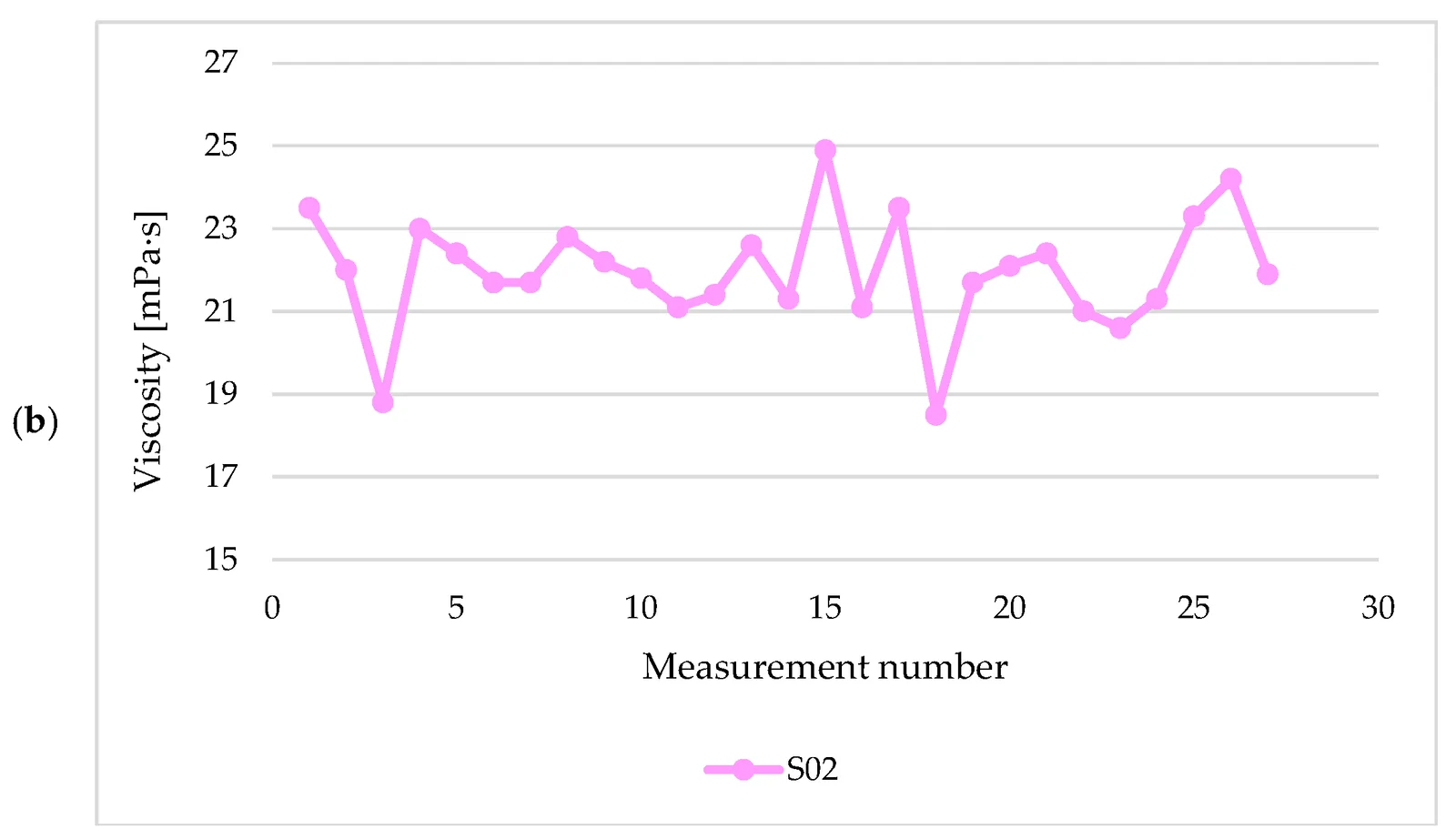
Changes in viscosity properties over time for (**a**) shampoo S01 and (**b**) shampoo S02.

For both car shampoo products, viscosity remained within a consistent range throughout the study, as shown in Figure 3. For shampoo S01, this parameter ranged from 25.1 to 31.7 mPa·s, while for shampoo S02, it ranged from 18.5 to 24.9 mPa·s. The values in both ranges were similar and did not indicate any significant changes in the product’s properties over time. The results indicate a stable composition for both developed products.

#### 2.5.3. Mass Loss

During the three-month stability tests conducted at elevated temperatures, no significant mass loss was observed in any analyzed samples of either shampoo. Mass changes over time remained stable and showed no upward trend. For shampoo S01, the average weight loss was 1.09 ± 0.00%, while for shampoo S02 it was 1.15 ± 0.01% over the entire test period. The observed weight loss was negligible and most likely resulted from partial water evaporation in the formulation. This change did not significantly affect the product’s performance, as the tested formulations are concentrates intended for further dilution with water before use.

#### 2.5.4. Changes in Product Turbidity

**Figure 4:**
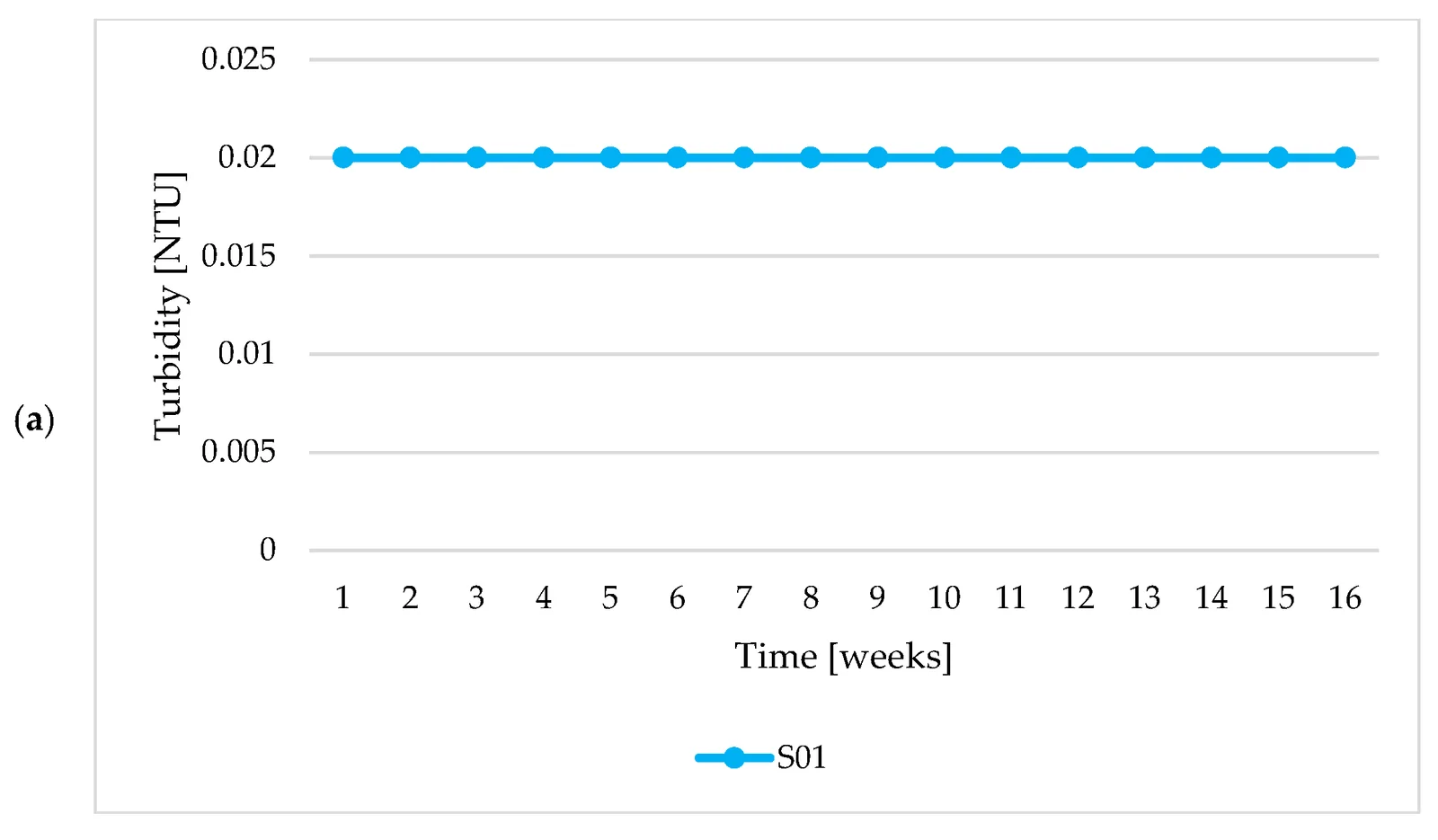

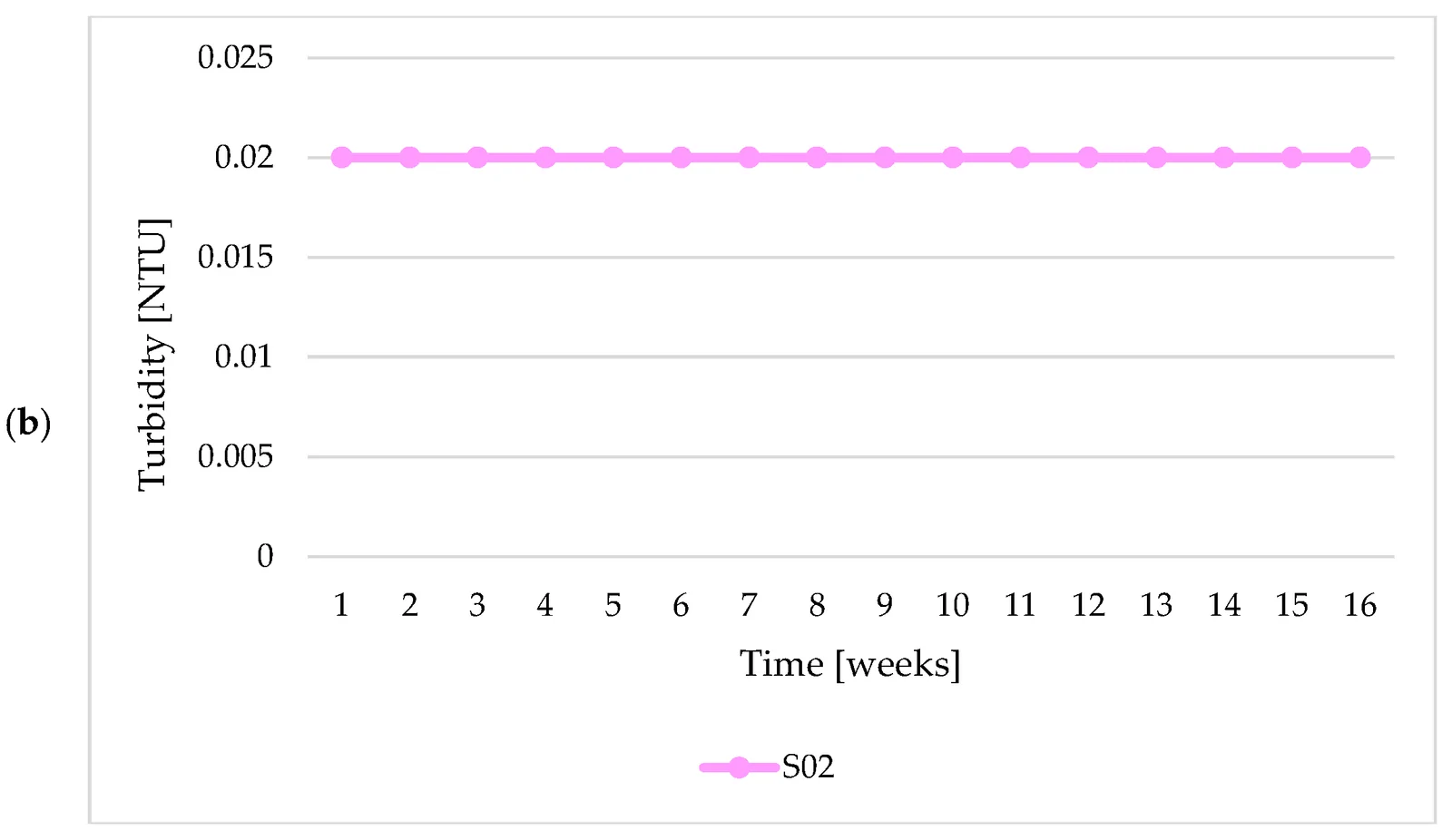
Changes in turbidity over time for: (**a**) shampoo S01; (**b**) shampoo S02.

No changes in turbidity were observed for either car shampoo over 3 months. Both shampoo S01 and shampoo S02 maintained a constant value of 0.02 NTU throughout the entire study period. Figure 4 presents the turbidity analysis results for both car shampoos. This result confirmed the theoretical assumption that, thanks to the high stability of the developed proprietary formulations, the turbidity parameter remains constant over time.

#### 2.5.5. Changes in Fluorescent Polymer Content

**Figure 5:**
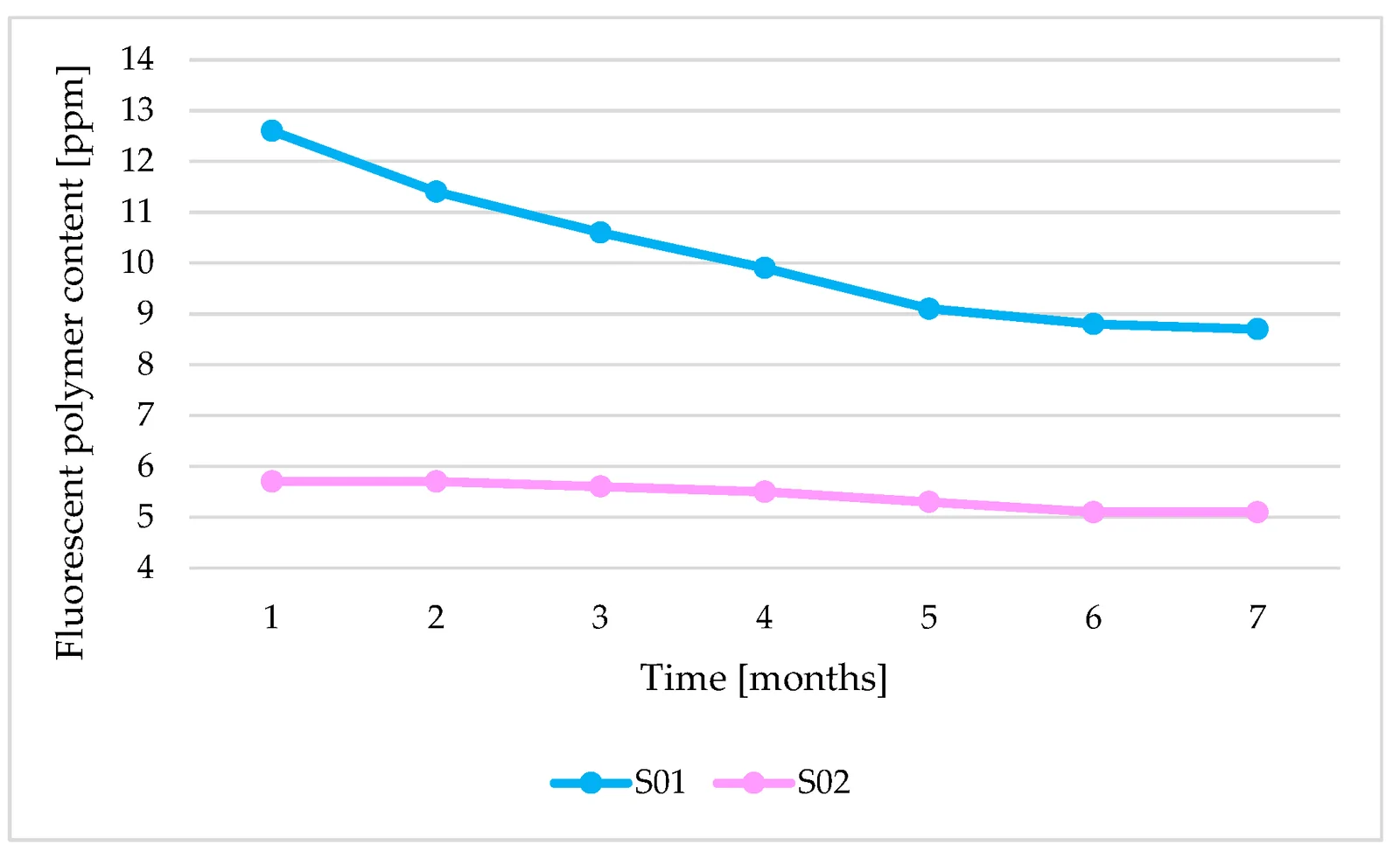
Changes in the concentration of fluorescent polymers in the test shampoo solutions over time.

During the fluorescent polymer content analysis, both shampoos showed a decrease in the measured parameter over time, but the trend differed between the proprietary shampoos. Shampoo S01 showed a more pronounced decrease, dropping from 12.6 ppm to 8.7 ppm. In contrast, shampoo S02 initially had a lower fluorescent polymer content (5.7 ppm), and the decrease in subsequent months was slight, reaching 5.1 ppm. Figure 5 shows a detailed presentation of changes in the tested parameter. Analysis of the last three months of the study showed that the decrease in polymer content was significantly smaller than in the initial period, particularly for shampoo S01. In the last two months of observation, the values obtained for both products were virtually identical or very similar.

### 2.6. Consumer Evaluation Results

**Figure 6:**
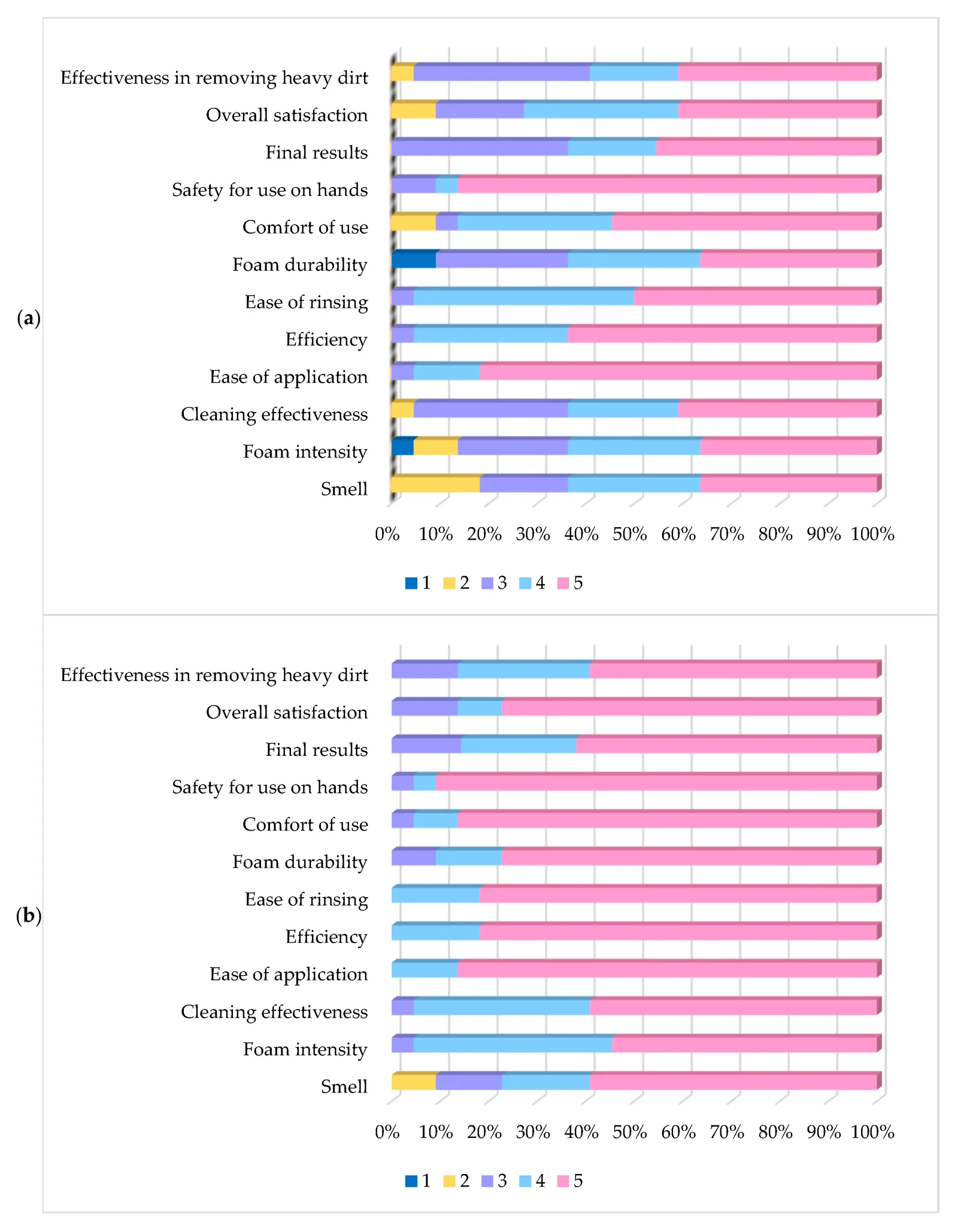

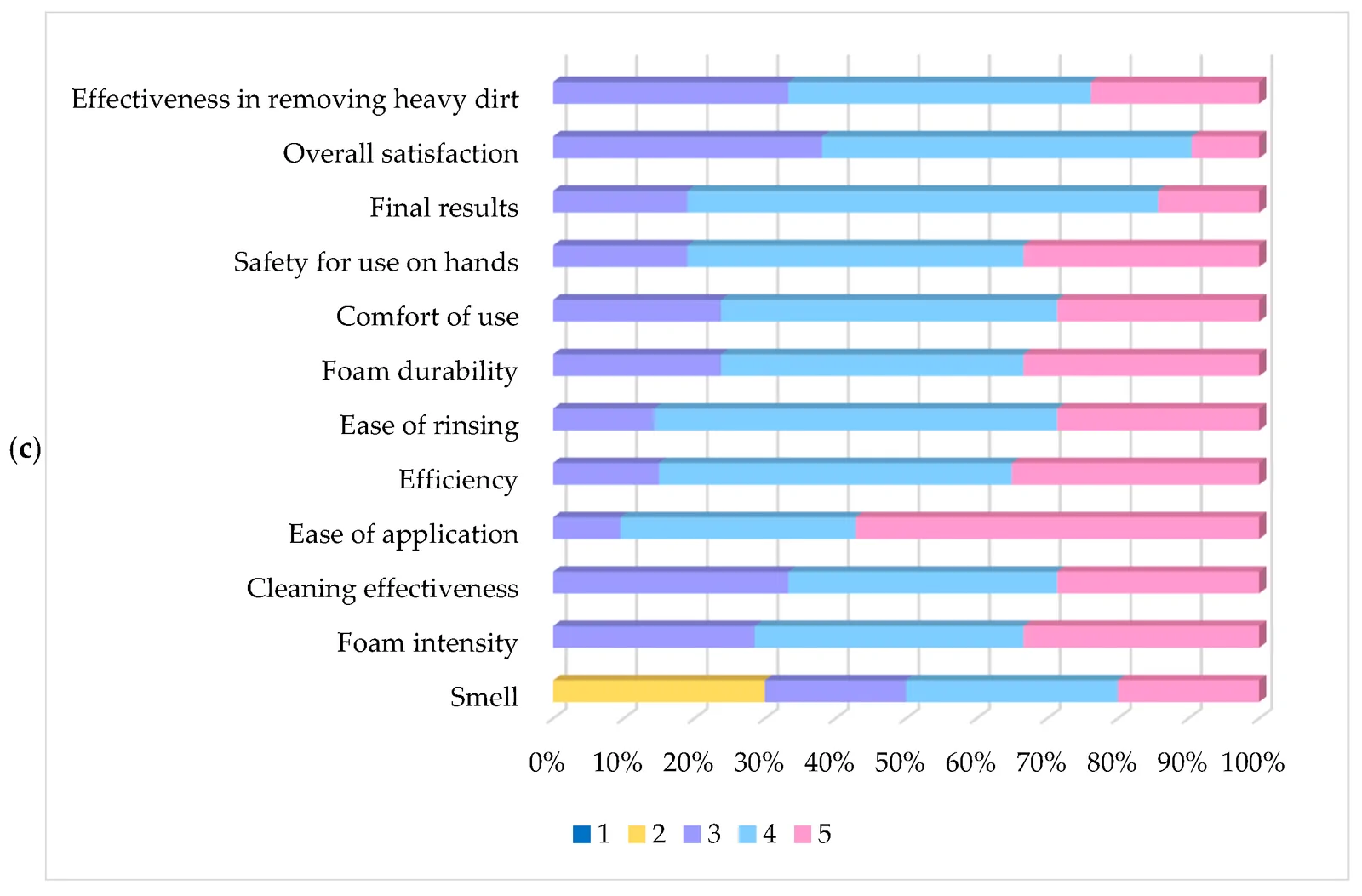
Survey results for the car shampoos tested: (**a**) Shampoo 1; (**b**) Shampoo 2; (**c**) Shampoo 3.

**Figure 7:**
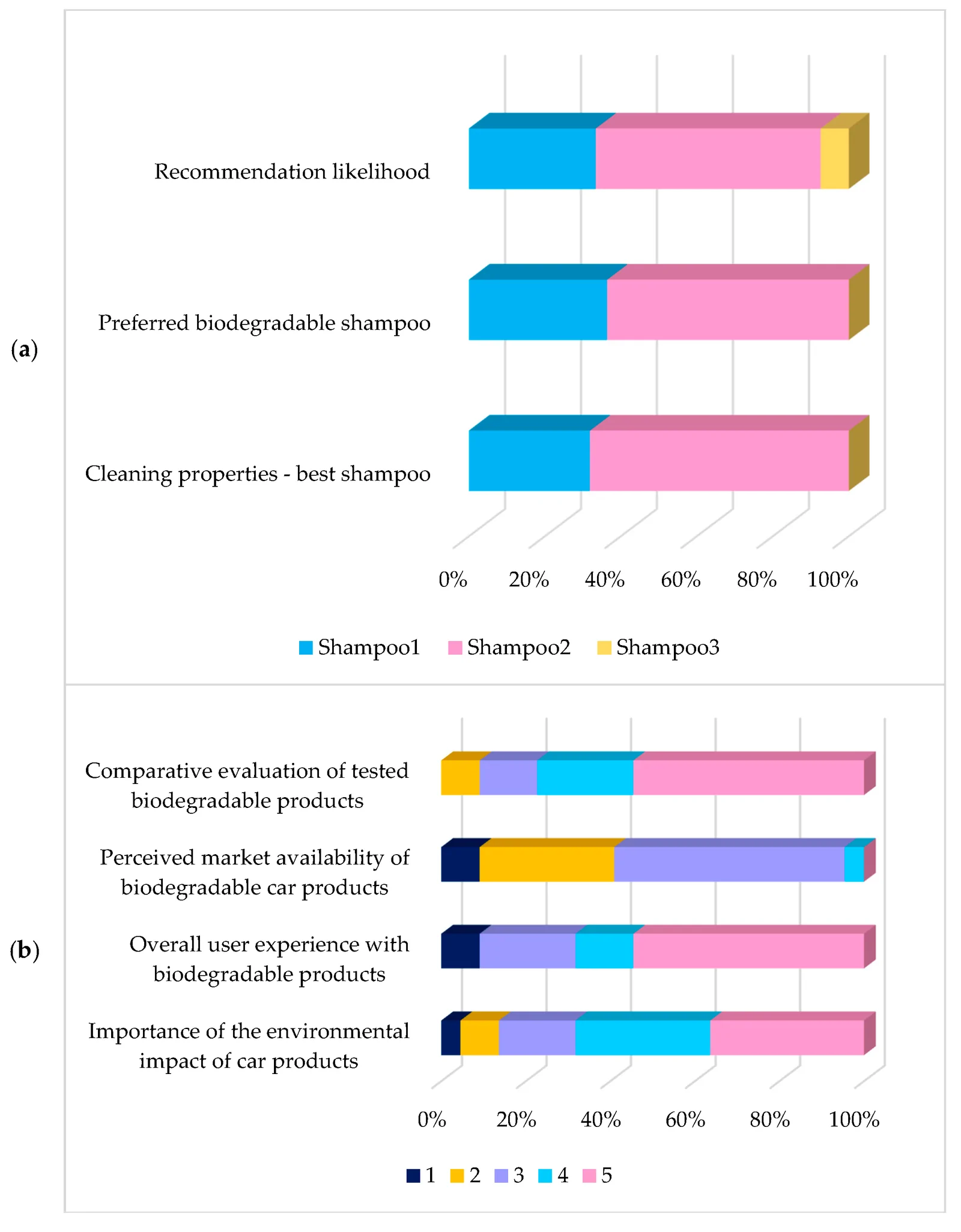
Results of the general section of consumer surveys regarding the car shampoos tested: (**a**) overall evaluation of the tested shampoos; (**b**) evaluation of biodegradable shampoos and the importance of environmental aspects.

The survey results are presented for both the general section, which characterizes the study participants and their attitudes toward environmental issues, and the individual car shampoos tested. Figure 6 presents the detailed analysis of the shampoos, while Figure 7 presents the general section.

Shampoo 2 received the highest ratings among the three products analyzed in all evaluation categories. The dominant rating in each category was the highest score (5), accounting from 50% to 90% of all responses, with the second most frequently selected rating being 4. Combined, the two highest ratings exceeded 80% of all responses for each criterion. Shampoo 1 also recorded a significant proportion of the highest ratings across all survey questions, though this proportion was lower than for Shampoo 2. At the same time, a clear proportion of positive and neutral responses was observed, and a small proportion of negative ratings appeared in some questions. For Shampoo 3, positive ratings constituted the largest share of responses, though not the highest. The highest and neutral ratings occurred in similar proportions. Negative responses appeared only for the question about the product’s scent. Analysis of the survey results indicates that participants rated Biodegradable Shampoo 2 as the best among the tested products, ahead of Biodegradable Shampoo 1 and Shampoo 3. The results may yield comparable, and in some respects even better, outcomes in consumer analysis than competing products.

The statistical analysis confirmed that the consumer evaluation of the three shampoo formulations differed significantly. The Friedman test showed a strong product effect (*p* = 0.000033), as reflected by the high Kendall’s *W* value of 0.861. This value suggests that product rankings were highly consistent across the evaluated criteria. Across these 12 criteria, the mean score was 4.20 for Shampoo 1 (S01), 4.65 for Shampoo 2 (S02), and 4.01 for Shampoo 3 (S11). The proportion of high ratings (scores 4–5) was 92% for Shampoo 2, compared with 76% for Shampoo 1 and 74% for Shampoo 3. The maximum score of 5 was also assigned most frequently to S02 (73%), followed by S01 (51%) and S11 (29%). The post-hoc analysis showed that Shampoo 2 differed significantly from both Shampoo 1 and Shampoo 3 (*p* = 0.0015 in both comparisons). This confirms that the higher consumer acceptance of S02 was statistically supported. The lack of a significant difference between Shampoo 1 and Shampoo 3 (*p* = 0.0630) indicates that consumers perceived these two formulations similarly, despite some numerical differences in individual criteria. The favorable performance of Shampoo 2 may be associated with its balanced sensory and functional properties. In particular, higher scores for foam-related parameters, comfort of use, ease of rinsing, and overall satisfaction suggest consumers perceived this formulation as more effective and more pleasant to use. The results represent a criterion-level comparison of product evaluation profiles rather than a full respondent-level repeated-measures analysis. Nevertheless, Shampoo 2’s highest rank across all 12 criteria provides strong evidence of a consistent and practically relevant improvement in consumer perception compared with Shampoo 1 and Shampoo 3.

The general section of the survey also included questions regarding participants’ attitudes toward environmental protection and consumer preferences. The responses confirmed the importance of environmental considerations and a significant portion of respondents’ interest in seeking more environmentally friendly alternatives. In addition, 19 participants stated they were willing to purchase biodegradable car shampoo in the future. In comparison, three participants did not provide a clear answer, stating they had not yet decided whether to make a purchase.

## 3. Discussion

The conducted research demonstrates the practicability of developing concentrated car shampoo formulations that combine high biodegradability with the physicochemical, microbiological, and functional properties required for practical use. The main scientific and practical value of the present study lies in the integrated evaluation of the developed formulations, including biodegradability, surface activity, microbiological protection, chemical composition, storage stability, packaging compatibility, and consumer acceptance. This comprehensive approach is particularly relevant for car cleaning products, as their environmental performance cannot be considered independently of their functional effectiveness and stability during use and storage.

The complete biodegradation observed for both formulations under the applied test conditions is particularly relevant from an environmental perspective. Car cleaning products are directly associated with the generation of wastewater containing surfactants and other potentially problematic contaminants, including oils, hydrocarbons, suspended solids and metals [27,34]. The demonstrated ready biodegradability therefore represents an important advantage of the developed products, as it indicates that their organic components can undergo biological degradation under the conditions defined by the applied standardized test. Importantly, the environmental advantage observed in this study was not achieved at the expense of the basic functional properties of the formulations. This supports the idea that highly biodegradable raw materials can be incorporated into the design of effective car cleaning products rather than treated as a separate formulation objective.

The surface tension results further support this interpretation. The developed products exhibited properties comparable to, or more favorable than, those observed for the commercial products included in the study. However, the similar surface tension values obtained for S01 and S02 at the tested concentration do not support a direct relationship between surface activity and the superior consumer acceptance observed for S02. The obtained results are consistent with previous reports showing that formulations based on bio-based or more environmentally favorable raw materials can retain effective wetting and cleansing properties [1,37]. From a formulation perspective, these findings suggest that a cleaning product’s environmental profile does not necessarily require a compromise in its fundamental surface-active performance. Instead, selecting and combining raw materials appropriately may allow environmental and functional requirements to be addressed simultaneously. This is an important consideration in developing more sustainable car cleaning products, where reduced environmental impact should be matched by adequate cleaning performance.

The microbiological stability of the developed formulations is also important for their practical applicability. The effectiveness of the preservative systems shows that using biodegradable raw materials does not inherently preclude adequate microbiological protection. Preservative efficacy should, however, be considered as a property of the formulation as a whole rather than attributed exclusively to the presence of individual preservatives. Factors such as formulation composition, water activity and storage conditions may contribute to the overall microbiological stability of cosmetic and related formulations [14,38]. The present findings therefore support the importance of considering the physicochemical environment created by the complete formulation when designing biodegradable concentrates with adequate microbiological protection.

The chromatographic analyses provide complementary evidence regarding the chemical integrity of the developed formulations. The observed profiles were consistent with the expected composition of systems containing ethoxylated surfactants. At the same time, no signals suggesting unexpected compounds or detectable degradation products were identified under the applied analytical conditions [39]. The agreement between the HPLC-MS and GC-MS findings and the formulation specifications is particularly relevant because it indicates that highly biodegradable raw materials can be used while maintaining satisfactory chemical integrity in the final products. Although the chromatographic analyses were qualitative and non-target, the absence of signals beyond those attributable to the declared formulation ingredients supports the chemical consistency of the developed products under the applied analytical conditions.

The stability and packaging assessment should be interpreted in the context of the overall physicochemical performance of the formulations. The absence of relevant changes in the evaluated physicochemical characteristics indicates that the developed concentrates maintained their overall quality under the applied storage conditions. This is consistent with general principles of stability assessment, which interpret changes in individual parameters in relation to the product’s overall quality profile rather than considering them independently [38,39]. The observed changes in the fluorescent polymer content may indicate potential interactions within the formulation-packaging system; however, the available data do not allow their origin to be determined conclusively.

The consumer evaluation adds a practical dimension to the findings. The favorable perception of the proprietary formulations, particularly the stronger overall consumer response to shampoo S02, indicates that incorporating environmental attributes into the formulation did not prevent the products from achieving satisfactory acceptance. The better performance of S02 may be related, at least in part, to differences in the characteristics of the surfactant raw materials used in the formulations. Although the nominal concentration of Capryl/Capryl Glucoside was identical in both formulations, the raw material used in S02 contained a higher active matter content (58–62%) than that used in S01 (40–44%). This difference may increase Capryl/Capryl Glucoside’s contribution to the overall surfactant activity of S02 and may therefore contribute to its more favorable functional performance and consumer acceptance. However, because the formulations also differed in the qualitative selection of other raw materials, S02’s superior performance should be attributed to the overall surfactant system rather than exclusively to Capryl/Capryl Glucoside. The research shows that the practical success of more sustainable cleaning products depends not only on their environmental characteristics but also on whether users perceive them as effective and convenient alternatives to conventional products. The results therefore suggest that appropriate formulation design can address environmental performance and consumer acceptance at the same time.

The high level of environmental awareness observed among the participants further emphasizes the practical relevance of developing biodegradable alternatives in this product category. Respondents’ declared willingness to consider biodegradable car shampoos aligns with previous observations that environmental awareness and product knowledge increasingly influence consumer evaluation of so-called green consumer chemicals [40]. Although consumer preferences cannot establish a formulation’s environmental value on their own, combining them with experimentally demonstrated biodegradability offers an important perspective for implementing more sustainable car cleaning products.

Taken together, the findings of this study highlight the potential of an integrated formulation approach for developing more sustainable car cleaning products. The developed concentrated shampoos combine highly biodegradable raw materials with the physicochemical, microbiological and functional properties required for practical application, while also demonstrating satisfactory storage stability and consumer acceptance. An important aspect of the present work is therefore considering biodegradability alongside product performance and practical applicability, rather than treating environmental performance as an isolated formulation characteristic. From an applied perspective, this approach provides a basis for further developing environmentally responsible car shampoo concentrates and highlights the importance of balancing environmental performance with product functionality, stability, packaging requirements, and consumer expectations.

## 4. Materials and Methods

### 4.1. Characteristics of Tested Car Shampoos

The study used two original formulations of biodegradable car shampoo concentrates (S01, S02). The formulations were developed using raw materials that meet the biodegradability criteria specified in OECD Test Guideline 301 [34]. The study aimed to develop products that combine high cleaning efficacy with a limited environmental impact. The tested formulations were prepared in the laboratory of the Faculty of Chemistry at Adam Mickiewicz University in Poznań (Poznań, Poland). The compositions of the tested products are presented below.

Shampoo S01—Ingredients: Aqua, C8–10 Alcohol Ethoxylate (2 EO), Caprylyl/Capryl Glucoside (raw material containing 40–44% active matter), Cocamidopropyl Betaine, 3-Methoxy-3-methyl-1-butanol, Glycerin, Trisodium Dicarboxymethyl Alaninate, Phenoxyethanol, Sanolin Lave Green G liquid VP 5225, Parfum.

Shampoo S02—Ingredients: Aqua, C8–10 Alcohol Ethoxylate (2 EO), Caprylyl/Capryl Glucoside (raw material containing 58–62% active matter), Cocamidopropyl Betaine, 3-Methoxy-3-methyl-1-butanol, Glycerin, Trisodium Dicarboxymethyl Alaninate, Phenoxyethanol, C.I. Acid Violet 126, Parfum.

The S01 and S02 formulations used the same quantitative composition, with identical nominal concentrations of the formulation components. The formulations differed qualitatively in the selection of surfactant systems, as well as in the colorant and fragrance used. Importantly, each corresponding component was incorporated at the same nominal concentration in both formulations; therefore, the differences between S01 and S02 result from the type and characteristics of the selected raw materials rather than from differences in their nominal concentrations. Detailed information concerning the chemical characteristics and suppliers of the raw materials used in the formulations has been reported previously [1].

For comparison, we also analyzed selected commercially available car shampoos (S11, S14, and S23), which were discussed in more detail in an earlier paper [1]. We used these products primarily to compare surface tension. Shampoo S11 was also used for comparative consumer evaluation tests. Table 1 presents the basic physicochemical properties of the shampoos used.

### 4.2. Evaluation of the Preservative Efficacy of the Shampoos

The efficacy of the preservative system in proprietary car shampoos was evaluated using a stress test method adapted from the PN-EN ISO 11930:2019-03 standard, including Amendment A1:2023-02 [14]. The reduction in microbial count (R_x_) was calculated using the following formula:

$$R_{x}=lgN_{0}-lgN_{x}$$

N_0_ is the number of microorganisms counted immediately after inoculation of the T_0_ sample, while N_x_ is the number of microorganisms counted at time T_x_ (7, 14, or 28 days). The initial number of microorganisms, N, was defined as the number of microorganisms in the inoculum, where N_0_ = N/100. No microbial growth (NI) at both 14 and 28 days was defined as no increase in the number of microorganisms compared with the previous time point. Efficacy was assessed in relation to criteria A specified in the PN-EN ISO 11930 standard [14].

### 4.3. Biodegradability Assessment

The biodegradability of proprietary car shampoos was assessed using the OECD 301B method, which is used for a screening assessment of a substance’s susceptibility to biodegradation under aerobic conditions in an aquatic environment. In the study, samples were incubated in a mineral substrate inoculated with a microbial culture, and then CO_2_-free air was passed through the system. The test was conducted for 28 days, in the dark or under diffused light. The degree of biodegradation was determined based on the amount of carbon dioxide released during the mineralization of the test sample by microorganisms. The released CO_2_ was captured in a suitable absorption solution, and the results were corrected for the blank value. The percentage of biodegradation of the products was calculated as the ratio of the measured CO_2_ evolution to the theoretical amount of CO_2_ that could be obtained upon complete mineralization of the test sample (theoretical CO_2_ production, ThCO_2_). According to OECD 301B, achieving at least 60% of ThCO_2_ within the specified test period is considered the pass level for ready biodegradability [34,35].

### 4.4. Surface Tension Analysis

To measure surface tension-reducing ability, 5% (*w*/*w*) car shampoo solutions were used as a standardized concentration for comparative assessment of the tested formulations. The solutions were prepared by weighing 1.00 g of the test product and adding distilled water to a total mass of 20.00 g. Measurements were conducted under laboratory conditions at 20 ± 1 °C using an Attension Sigma 700 tensiometer (Biolin Scientific, Gothenburg, Sweden). The Wilhelmy plate method was used. The tests involved immersing a glass plate in the solution and then slowly withdrawing it while recording the force acting on its surface. Based on the recorded force values, the surface tension of the tested solutions was determined using the Wilhelmy method equation. For each solution analyzed, 10 measurements were performed, and the average value was calculated.

### 4.5. Chromatographic Analysis

A series of chromatographic analyses was conducted to assess the purity of the obtained products and to screen for additional compounds not expected from the declared formulation. In the first stage, high-performance liquid chromatography coupled with mass spectrometry (HPLC-MS, Waters HPLC-MS with an SQ Detector 2 (SQD-2), Waters, Milford, MA, USA) was used, followed by gas chromatography coupled with mass spectrometry (GC-MS, Varian GC-MS 4000, Walnut Creek, CA, USA). GC-MS analyses were performed after dissolving the samples in methanol and chloroform, with a single analytical run lasting 25 min. For HPLC-MS analyses, we used methanol-acetonitrile gradient elution, with the mobile phase composition changing linearly from 20:80 to 80:20 (*v*/*v*). The injection volume was 20 µL, and the total analysis time was 30 min, with a detection range of 100–1000 *m*/*z*. For GC-MS samples, shampoo sample S02 did not include the dye because it is soluble only in an aqueous medium, which could affect the analysis under gas chromatography conditions.

### 4.6. Consumer Evaluation

**Figure 8:**
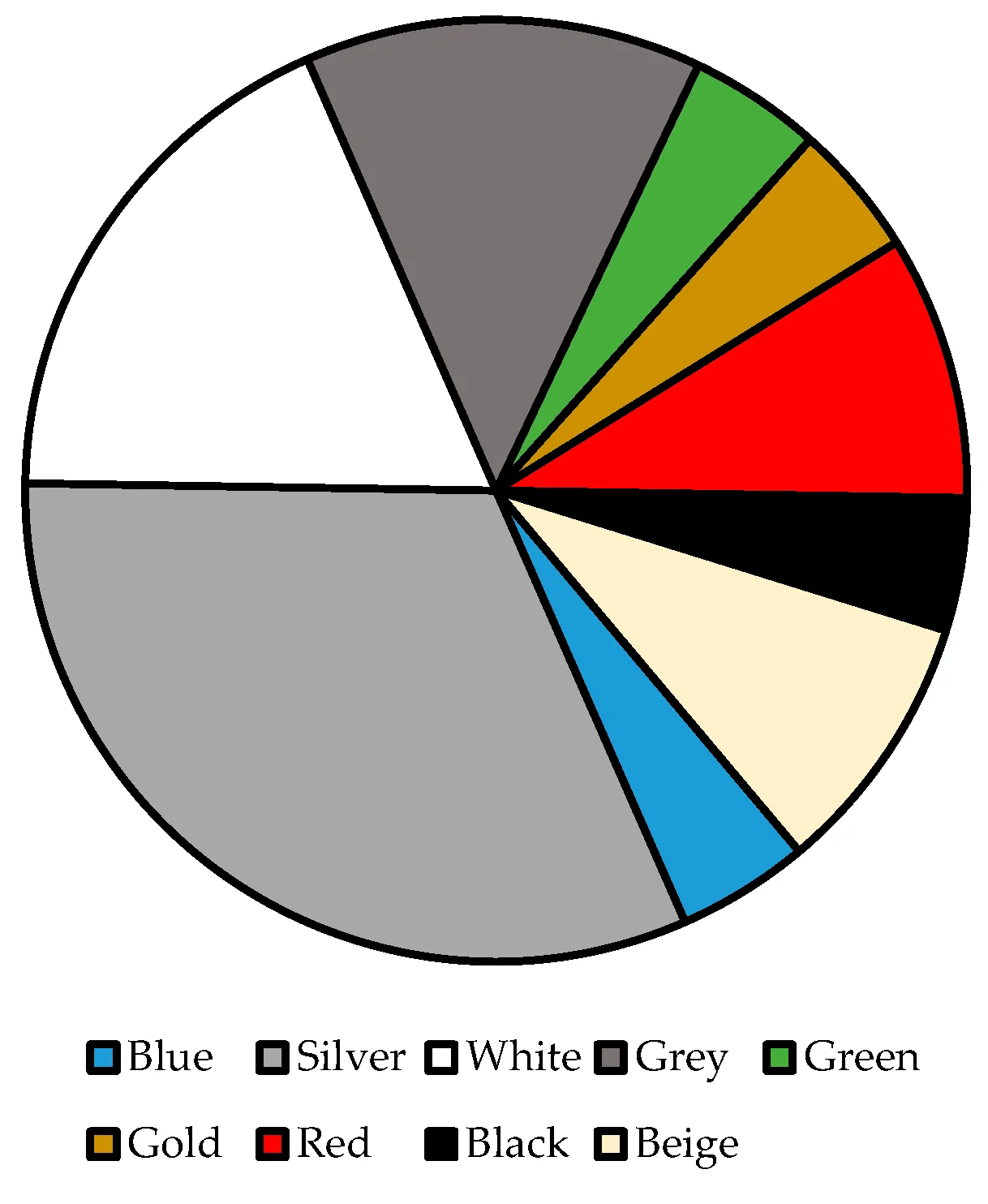
Types of car paint colors among participants in the consumer survey.

A total of 22 car owners were recruited for the study. The study group consisted of 14 men and 8 women representing various age groups: 3 people under 25; 9 people aged 26–35; 5 people aged 36–45; 2 people aged 46–55; and 3 people over 55. Based on the initial interview, it was determined that 16 of the 22 participants had not previously used a biodegradable car shampoo. In addition, when asked how often participants wash their cars, 5 people replied once a week, 11 once a month, and 6 participants less than once a month. Each participant signed an informed consent form to participate in the study. Figure 8 shows the paint colors on the participants’ cars where the tests were conducted.

Each participant received three 50-mL samples of car shampoos for testing. The sample set included:

• Shampoo 1—S01 (biodegradable);
• Shampoo 2—S02 (biodegradable);
• Shampoo 3—S11, a commercial car shampoo used for comparison purposes (non-biodegradable).

Additionally, participants were instructed on how to dilute the shampoos, the recommended concentration (1:200), the application rules, and the criteria for evaluating the products, including subjective assessment. The 1:200 dilution was selected as a practical application concentration based on previous studies showing comparable cleaning performance [1]. At the end, each participant completed an online survey with general questions and questions about each tested shampoo.

#### Statistical Analysis

Consumer survey data were statistically analyzed using Statistica 13.3 (StatSoft, Krakow, Poland). Ratings were recorded on a five-point Likert scale, where 1 indicated the lowest score and 5 the highest score. Weighted mean scores were calculated for each shampoo and each evaluation criterion. The product profiles were compared using the non-parametric Friedman ANOVA by ranks, treating evaluation criteria as repeated blocks and shampoo type as the tested factor. Differences were considered statistically significant at *p* < 0.05. When significant differences were found, post-hoc pairwise comparisons were performed using the Wilcoxon signed-rank test. The Bonferroni correction was applied for multiple comparisons, and the adjusted significance threshold for post-hoc tests was set at *p* < 0.0167. The strength of the product effect was expressed as Kendall’s coefficient of concordance *W*.

### 4.7. Assessment of Product-Packaging Compatibility

The original products underwent product-packaging compatibility analysis. Both shampoos were placed in white, 750-mL bottles made of recyclable PET plastic. For each shampoo, a set of three bottles was prepared for testing. After baseline analysis, the samples were placed in an incubator set to 40 °C for three months and stored upright. The temperature of 40 °C was selected as an elevated storage condition to accelerate potential physicochemical changes and possible interactions between the formulations and the packaging material that may occur during prolonged storage. The three-month exposure period was selected to provide sufficient time to monitor potential changes in the investigated product and packaging-related parameters under these conditions. The applied conditions were therefore intended to detect potential changes rather than to represent a specific period of storage at room temperature directly. Twice a week, the samples were measured for changes in pH and viscosity, while once a week, the samples were analyzed for weight loss and turbidity. Additionally, samples of shampoo solutions with a concentration of 13.33% were prepared and stored in a dark room at room temperature. For the 100% shampoo samples, results that exceeded the instrument’s measurement range were recorded during the determination of fluorescent polymer content. Consequently, a decision was made to conduct the tests using shampoo solutions, which allowed for a more accurate analysis of potential changes occurring in the packaging material. The fluorescent polymer content in the prepared samples was analyzed over seven months, with monthly measurements. All tests were conducted under laboratory conditions at room temperature.

#### 4.7.1. pH Analysis

pH values were measured using the EcoSense^®^ pH 10 pH/Temperature Meter, Pen Style (VWR International, Radnor, PA, USA). For each shampoo tested, measurements were taken twice a week, with three replicates each time, and the average pH value was then calculated.

#### 4.7.2. Viscosity Analysis

The viscosity of the proprietary shampoos was determined using an IKA ROTAVISC me-vi viscometer (IKA Poland Sp. z o.o., Warsaw, Poland) equipped with a VOL-SP-6.7 spindle (IKA Poland Sp. z o.o., Warsaw, Poland). Samples of the tested product with a volume of 6.7 cm^3^ were collected for analysis and then analyzed in automatic mode. Measurements were performed in triplicate at room temperature twice a week over three months, and the results were averaged.

#### 4.7.3. Mass Loss Analysis

Changes in weight loss were monitored throughout the study period in samples stored in sealed bottles. Measurements were taken weekly using a Sartorius BP 2100 S scale (Sartorius, Göttingen, Germany), and the average value was calculated each time from three replicate measurements. Only the product weight was analyzed, excluding the bottle and cap.

#### 4.7.4. Shampoo Turbidity Analysis

The turbidity of the car shampoos was analyzed using a T10 Turbidity Meter (MANTECH, Guelph, ON, Canada). Measurements were taken at room temperature once a week over three months. Samples of 30 mL of each car shampoo were used for the analysis. Each measurement was performed in triplicate, and the final results were averaged.

#### 4.7.5. Fluorescent Polymer Content Analysis

The analysis of 13.33% car shampoo solutions was performed using a Pyxis SP-350P analyzer (Pyxis Lab, Tomball, TX, USA). This device enables rapid measurement of fluorescent polymer content while minimizing interference from factors such as sample color or turbidity. The analyzed parameter was determined in the 0 to 20 ppm concentration range. The measurement involved placing a sample of the tested solution with a volume not exceeding 5 mL in the measurement chamber, followed by reading the result. The tests were conducted at room temperature, with measurements taken once a month over a period of seven months. Each time, a series of three measurements was performed, from which the average value was calculated. The solution samples were stored in a cool place with limited light exposure.

## 5. Conclusions

Among all analyses, Shampoo S02 performed particularly well, combining excellent biodegradability, high microbiological protection efficacy, stability in packaging compatibility tests, and the highest consumer acceptance. Shampoo S01 also showed favorable properties; however, in some tests—such as usability assessment and fluorescent polymer content—its profile was less favorable than S02. These results indicate that both developed products have application potential, with S02 being the more optimal solution in terms of simultaneously meeting environmental, technological, and consumer criteria.

## Figures and Tables

**Table 1:** Basic physicochemical properties of car shampoos used in the study [1].

Sample	pH (±SD)	Density (±SD) [g/cm^3^]	Viscosity (±SD) [mPa·s]
S01	7.00 ± 0.01	1.0081 ± 0.0054	26.5 ± 0.00
S02	7.24 ± 0.01	1.0142 ± 0.0133	22.3 ± 0.14
S11	9.64 ± 0.04	1.0277 ± 0.0049	-
S14	4.87 ± 0.07	0.9898 ± 0.0069	82.3 ± 11.10
S23	6.58 ± 0.04	1.0054 ± 0.0015	-

## Data Availability

The original contributions presented in this study are included in the article/Supplementary Materials. Further inquiries can be directed to the corresponding author.

## Supplementary Materials

The following supporting information can be downloaded at https://www.mdpi.com/article/10.3390/ijms27188050/s1.

## Abbreviations

The following abbreviations are used in this manuscript:

CMC	Critical Micelle Concentration
HPLC-MS	High-Performance Liquid Chromatography-Mass Spectrometry
GC-MS	Gas Chromatography-Mass Spectrometry
