# Supplementary material for: Characteristics of Biodegradable Car Shampoos in Terms of Performance and Environmental Properties
Source: Int J Mol Sci. 2026 Sep 10;27(18):8050. doi: 10.3390/ijms27188050 (PMC13606842; doi:10.3390/ijms27188050)
Supplement: Supplementary file 1 [file ijms-27-08050-s001.zip › ijms-4510682-supplementary.pdf]

Supplementary Materials

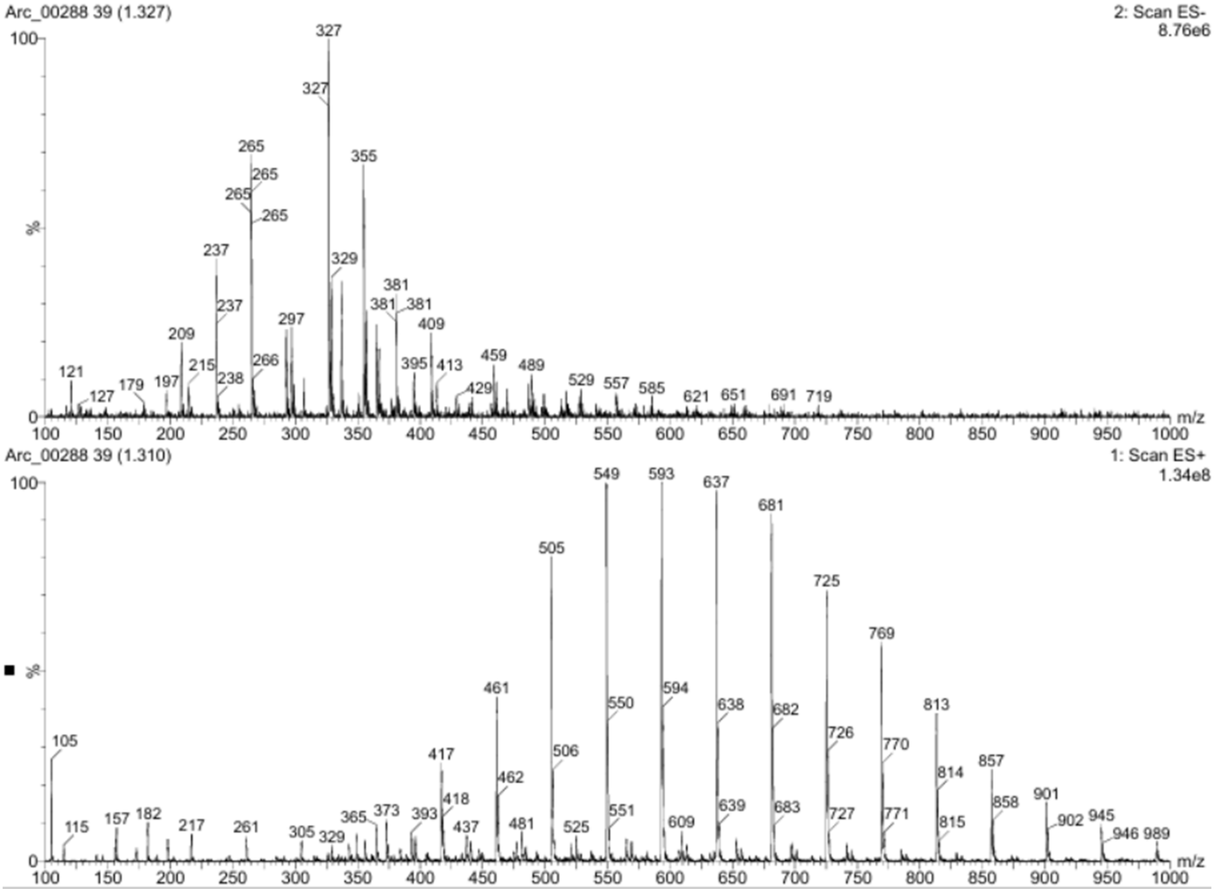

Figure S1. Chromatographic analysis of shampoo S01 obtained using HPLC-MS.

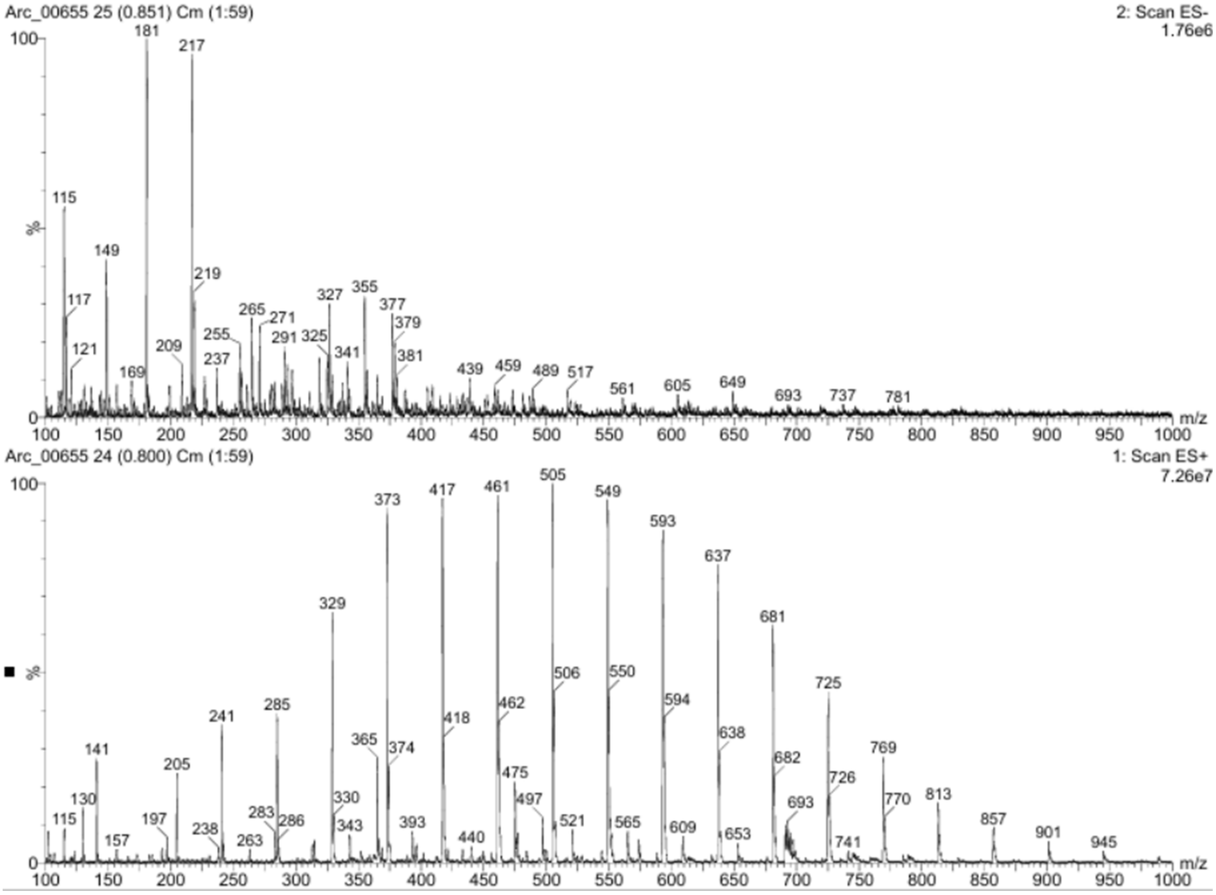

Figure S2. Chromatographic analysis of shampoo S02 obtained using HPLC-MS.

(a)

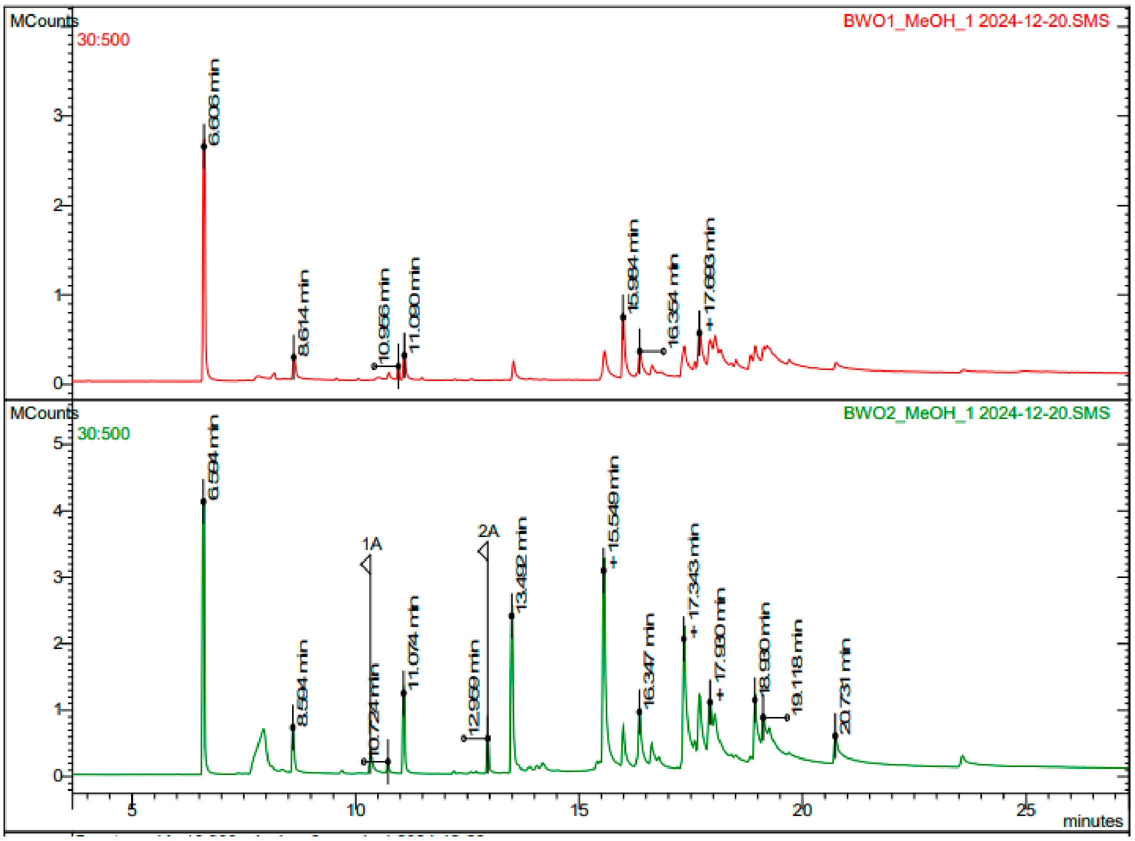

(b)

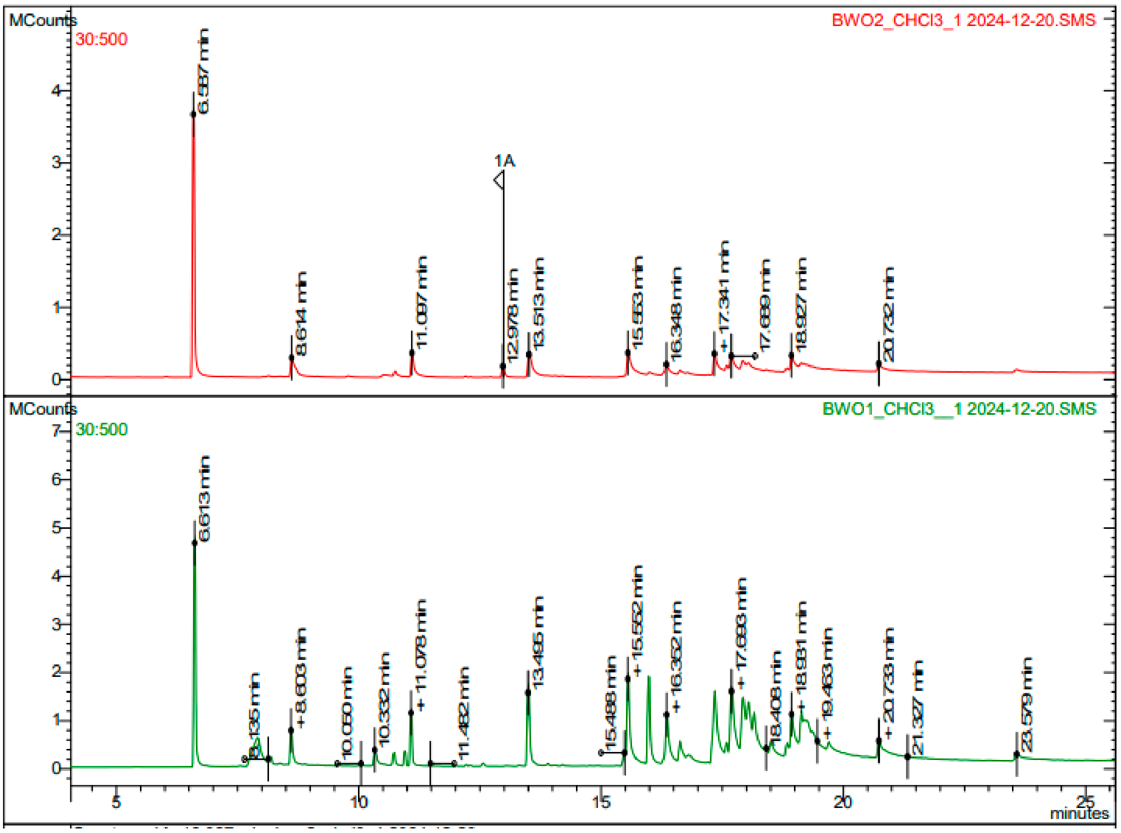

Figure S3. Gas chromatography coupled with mass spectrometry analysis of proprietary car shampoo samples dissolved in a) methanol and b) chloroform. (For BW01 = S01; BW02 = S02).
